# The Nonradiographic Axial Spondyloarthritis, the Radiographic Axial Spondyloarthritis, and Ankylosing Spondylitis: The Tangled Skein of Rheumatology

**DOI:** 10.1155/2017/1824794

**Published:** 2017-05-07

**Authors:** Anand N. Malaviya, Roopa Rawat, Neha Agrawal, Nilesh S. Patil

**Affiliations:** Department of Rheumatology, The Joint Disease Clinic, ISIC Superspeciality Hospital, Vasant Kunj, New Delhi 110070, India

## Abstract

Since 1984 the diagnosis of ankylosing spondylitis (AS) has been based upon the modified New York (mNY) criteria with mandatory presence of radiographic sacroiliitis, without which the diagnosis is not tenable. However, it may take years or decades for radiographic sacroiliitis to develop delaying the diagnosis for long periods. It did not matter in the past because no effective treatment was available. However, with the availability of a highly effective treatment, namely, tumour necrosis factor-*α* inhibitors (TNFi), the issue of early diagnosis of AS acquired an urgency. The Assessment of SpondyloArthritis International Society (ASAS) classification criteria published in 2009 was a significant step towards this goal. These criteria described an early stage of the disease where sacroiliitis was demonstrable only on MRI but not on standard radiograph. Therefore, this stage of the disease was labelled “nonradiographic axial SpA” (nr-axSpA). But questions have been raised if, in search of early diagnosis, specificity was compromised. The Federal Drug Administration (FDA, USA) withheld approval for the use of TNFi in patients with nr-axSpA because of issues related to the specificity of these criteria. This review attempts to clarify some of these aspects of the nr-axSpA-AS relationship and also tries to answer the question whether ASAS classifiable radiographic axial spondyloarthritis (r-axSpA) term can be interchangeably used with the term AS.

## 1. Introduction

**Figure 1:**
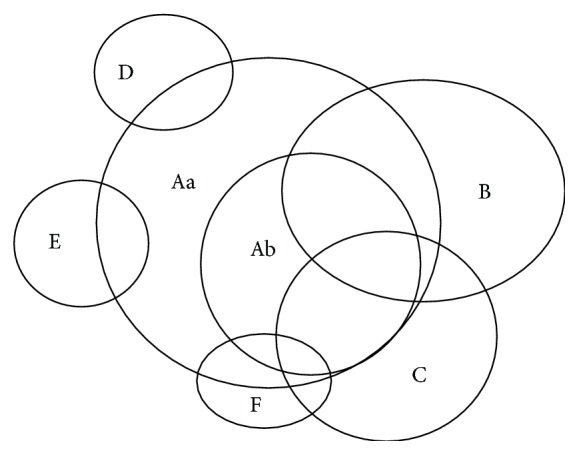
The circles “A,” “B,” and “C” represent (1) the axial spondyloarthritis (axSpA); (2) the peripheral SpA, and (3) the extra-articular manifestations of SpA, respectively. Within the axSpA (circle A) the two subcomponents are “Aa” and “Ab” that represent nonradiographic axSpA (nr-axSpA) and radiographic axSpA (r-axSpA), respectively. Circles “B,” “C,” “D,” “E,” and “F” represent (i) peripheral arthritis of SpA pattern, (ii) extra-articular features, (iii) inflammatory bowel disease-related SpA, (iv) psoriasis-related SpA, and (v) reactive arthritis-related SpA. Patients in circle “A” are often identified as* “primary”* SpA and those in circles “C,” “D,” or “E” as* “secondary”* SpA.

In today's terminology, primary inflammatory disease of the spine is called axial (ax) spondyloarthritis (SpA) or axSpA [1]. It has a strong genetic predisposition with a high proportion of patients carrying the HLA B27 genotype [2]. In Northern parts of India, the prevalence of this gene in general population is ~6% while that among patients with SpA is >90% [3]. The concept of spondyloarthritis or “SpA” evolved in the 1970s in the United Kingdom. Professor Wright's group at Leeds (UK) describes a closely related cluster of conditions with specific common clinical, epidemiological, genetic, and radiographic characteristics [4, 5]. It included ankylosing spondylitis (AS), the disease prototype, and psoriatic arthritis- (PsA-) related, inflammatory bowel disease- (IBD-) related, and reactive arthritis- (ReA-) related SpA. Among these entities it was not uncommon to see a characteristic form of peripheral arthritis affecting the joints in the extremities as well as the “root”/“central” joints that included sacroiliac joints (SIJ), pubic symphysis, and sternoclavicular and temporomandibular joints. The pattern of the peripheral arthritis was usually below waist, asymmetrical large joint involvement. The common extra-articular features included recurrent acute anterior uveitis (AAU), enthesitis, and dactylitis. Wright and colleagues called this entity “seronegative spondarthritis” (SSA). Over the years the nomenclature of this entity has changed. In 1991 the European Spondyloarthropathy Study Group (ESSG) gave the name “spondyloarthropathy” [6]. In the year 2002 a group of experts further modified it to “spondyloarthritis” (SpA), thus removing any ambiguity in the term “…  arthropathy” that may lead to the inclusion of noninflammatory spinal diseases [7]. The term SpA is now formally recognised for this spectrum of diseases. As mentioned above, there are three clinical components of SpA, namely, (1) axial (axSpA); (2) peripheral SpA (pSpA); and (3) extra-articular manifestations. These 3 components of SpA along with certain disease-defining subgroups of SpA are depicted in Figure 1.

Thus, SpA has been conceptualised as a condition with a broad spectrum of clinical manifestations, laboratory abnormalities, and imaging features [1]. Such broad array of clinical manifestations makes SpA a difficult disease to comprehend and recognise in clinical practice [8]. Historically, a clinical condition is always identified first in its most advanced stage when the manifestations are glaring. The most advanced stage of SpA with dramatic clinical manifestations (including the typical posture of a late case) is* ankylosing spondylitis *(AS). Therefore, AS was recognised and extensively written about many decades prior to the emergence of the concept of SpA [9]. The introduction of the concept of SpA then raised an important question;* “Is the axial component of SpA the same disease as AS?”* The issue has become a subject of much debate. This review discusses the relationship of axial SpA (axSpA) and AS taking in account the historical perspective including the old [10] and the new classification system [11] that has caused a raging controversy.

## 2. Historical Aspects

The terms “spondyl” and “spondylo” are Greek words for “vertebra,” and “itis” means inflammation. Similarly, “ankylosing” means “fusing together” [12], thus came the term ankylosing spondylitis or AS. The first description of what we recognise today as AS was by Bernard Connor (1666–1695) [9]. Without going into further historical details, it suffices to point out that, despite the name AS, the involvement of the sacroiliac joints (SIJ) detected on a standard posterior-anterior radiograph of the pelvis (sacroiliitis) was recognised as the most important factor for the diagnosis of AS [7]. Therefore, radiographic sacroiliitis became the sine qua non for this disease. For this reason, the modified New York (mNY) criteria for the* diagnosis *of AS included, besides inflammatory back pain, limitation of mobility of the lumbar spine and limitation of chest expansion (any one of the 3 clinical criteria), the radiographic criteria of definite sacroiliitis (minimum grade II bilaterally or grade III unilaterally), without which the diagnosis of AS was not tenable [7, 10].

During the late 1970s and early 1980s, while working at the All-India Institute of Medical Sciences (AIIMS), New Delhi, the senior author (ANM) had frequently observed fathers with advanced AS bringing their young sons complaining of back pain with features strongly suggestive of inflammatory nature [13]. However, the radiographs of their pelvis did not show sacroiliitis or showed only minimal changes that did not satisfy the mNY criteria. Based on these observations ANM's group at AIIMS reported for the first time in 1983 what was believed to be an early stage of AS where the radiographic sacroiliitis had not developed [14]. Because we could not label such patients as AS, we called this clinical entity* “unclassifiable spondyloarthritis.”* Seven years later, Professor Amor's group in France, deeply involved with the subject of AS research, described the spondyloarthritis spectrum of manifestations leading to the classification criteria for those who did not fit the mNY criteria [15]. Amor's group called this entity* “undifferentiated spondyloarthritis,”* the term that was accepted worldwide. A European team of specialists updated these criteria that came to be widely known as the ESSG criteria [6]. These reports strongly indicated that AS was very likely the late stage of “unclassifiable” or “undifferentiated” SpA. Both of these “early” and “late” clinical stages, however, had a similar spectrum of clinical features. These consisted of (i)* inflammatory back pain *[13, 16, 17]; (ii) a certain proportion having a rather characteristic* peripheral arthritis *affecting large joints in the lower segment of the body with prominent asymmetry and tarsitis [18]; (iii) characteristic* extra-articular features *consisting of episodes of acute anterior uveitis, prominent enthesitis, dactylitis, and several others features [19]. Thus, the concept evolved that AS (diagnosed with mNY criteria) was a late stage of a spectrum of inflammatory spinal disease. In the year 2002, a group of experts recommended the use of the term spondyloarthritis (SpA, indicative of its inflammatory nature) that was recommended against the term spondyloarthropathy suggested in ESSG criteria that had a connotation of being either inflammatory or noninflammatory disease [7]. In the early stages of this entity, the radiographic sacroiliitis could be absent because it would take years to develop these changes. Furthermore, this happens only in a proportion of patients, while others may not progress further [20, 21]. Then the question arose whether these early patients had a similar level of disease burden as determined by the severity of symptoms. Using the German Spondyloarthropathy Inception Cohort (GESPIC) the German workers showed that in patients with SpA (in the absence of radiographic sacroiliitis) the* “disease burden”* as determined by the severity of symptom (measured by the disease activity index {⁡the Bath Ankylosing Spondylitis Disease Activity Index⁡}, seriousness of global pain and night pain, patient's global assessment of disease activity, intensity of treatment, response to treatment, and quality of life), does not differ from the patients with radiographic sacroiliitis (i.e., mNY classifiable AS) of <10 years duration [22–24]. This would mean that these patients are as sick as patients with AS and deserve the same intensity of treatment for the symptom relief.

In those days such theoretical discussions had little practical clinical relevance because, whatever was the stage of the disease (early or late), there was hardly any effective treatment to halt the disease progression. Physical therapy and nonsteroidal anti-inflammatory drugs (NSAIDs) were the mainstays of treatment. These measures did provide symptom relief but only in a certain proportion with no certainty of halting the disease progression. There were no truly effective therapeutic choices.

## 3. Discovery of Tumour Necrosis Factor-***α*** Inhibitor Treatment and the Importance of Early Diagnosis

The pessimistic scenario of AS changed dramatically when Braun and colleagues in the year 1995 demonstrated the abundant presence of tumour necrosis factor alpha (TNF-*α*) on histopathology of active sacroiliac joints of these patients [23]. TNF-*α* inhibitor (TNFi) infliximab was already approved by the Federal Drug Administration (FDA), the USA, for the use of rheumatoid arthritis in November 1999 [25]. Several controlled trials of TNFi in AS between 2001 and 2003 confirmed its dramatic response that had never been seen in past with any intervention [26–34]. This singular discovery compelled the treating physicians to look at AS from a different standpoint. The reasoning was* “If a drug gives such a dramatic relief at a stage when damage is already advanced (radiographic sacroiliitis and osteoproliferation in the spine), won't it be much more reasonable to give the same treatment at an early stage of the disease”*? Naturally, a method for early diagnosis became an urgent need of the times. In the search for a method for early diagnosis of SpA it was essential to review the existing knowledge on the natural history of AS. From this standpoint, the advent of magnetic resonance imaging (MRI), and its use in studying SIJ, was a significant advance in understanding the disease [35–37]. It was found that patients with clinically suspected AS but without radiographic sacroiliitis showed exuberant juxta-articular active inflammatory lesions (by way of bone marrow oedema) in the SIJ [38]. Several studies had also reported a dramatic response to TNFi treatment that ameliorated active sacroiliitis seen on MRI [26, 34]. Workers had also demonstrated that the response to TNFi was much more impressive in those with short disease duration and with high acute phase reactants (ERS, CRP) [39, 40]. Two important points emerged from these early reports. First, the possibility that by suppressing inflammation using TNFi before structural damage occurs, could prevent long-term structural damage seen as radiographic progression [39]. Second, early diagnosis seemed to be at the centre of this approach because such patients showed better response [40].* Thus, an early and reliable diagnosis of AS became a central point for the appropriate management. *

## 4. Inherent Difficulties in the Early Diagnosis of AS: It Takes Years to Develop Radiographic Sacroiliitis but Only in Predisposed Individuals; in Some Patients, the Radiographic Changes May Never Develop

The studies on “unclassifiable”/“undifferentiated” form of AS have been already alluded to earlier. Some workers have reported on the disease progression and the development of the radiographic changes over time [20, 21, 41, 42]. The results of these studies can be summarised as follows: in a group of patients with <45 years of age, the radiographic sacroiliitis was present in 16% as against 38% in the other group >45 years of age, ostensibly having the disease for a longer time. In a study of patients with 10-year disease duration, radiographic sacroiliitis was present in 40%, in those with 10–19-year disease duration this was seen in ~70% and those with >20-year disease duration it was present in 86%. These observations establish that* symptom *or* disease duration is pivotal for the damage *that is the development of radiographic sacroiliitis. However, these studies also showed that a certain proportion of patients at an early stage of the disease neither progressed nor developed the radiographic changes in spite of the long duration of the disease. This observation is equally important and would mean that* besides duration there could be other factors involved in the development of damage *(i.e., radiographic sacroiliitis, chronic changes of erosions, and osteoproliferation including the development of syndesmophytes). In other words, disease progression is not only determined by the duration but also by additional* predisposing or risk factor(s)*.

## 5. Predisposing Factors for the Progression of the Disease towards Radiographic Sacroiliitis and Damage: The Continuum of AS

**Figure 2:**
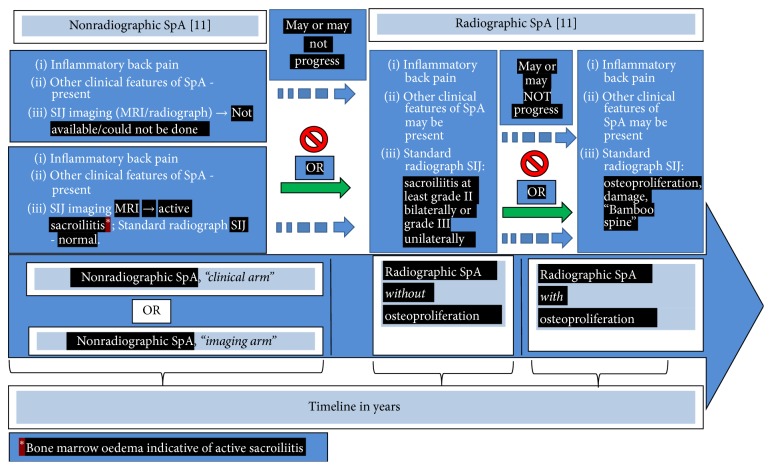
Natural history of axial SpA over timeline → from left to right. For details see text.

Over the years a number of risk factors that increase the chances of the progression from nonradiographic to the stage of radiographic damage in SIJ have been identified. The influence of* gender *has been recognised since the early description of AS, so much so that for a long time a myth was perpetuated that AS did not occur in women till the seminal work by Hart and Robinson who described AS in women in 1959 [43]. The authors emphasised that there was no significant difference in the clinical features between males and females except that, on the whole, it seems to be milder in the latter and less likely to produce extensive spinal changes. The authors mentioned that, for this reason, it was more likely to be overlooked in women. The male gender bias for the development of more aggressive disease and the mechanism involving Th-17 has been recently reported [44]. Additionally, there are some studies that have shown a predominance of males among those with radiographic sacroiliitis while the proportion of men and women is equal or may even show female preponderance among those without radiographic sacroiliitis [45–50]. Thus, male gender is a strong predisposing factor for the development of progressive disease. Another genetic influence is the* presence of HLA B27*. Several studies have shown a strong association of AS with HLAB27 positivity and much weaker association in patients without radiographic sacroiliitis [51]. Recently, several additional genetic factors (e.g., ERAP-1, IL23R) [52] and certain protein biomarkers have been found to influence the disease progression and radiographic damage. Thus, the KIR3DL1, a receptor protein biomarker, seems to have a protective effect against the most severe manifestations of AS [53]. Pathologic bone formation in AS might be due to molecular dysfunction of certain other protein biomarkers, for example, Sclerostin and Dkk-1, at the cellular level [54].* Age of symptom onset *is another well-described predisposing factor for progressive disease; the earlier the symptom onset is, the more likely it is to be progressive [55, 56]. Smoking, lifestyle factors, and other socioeconomic/environmental factors including a physically demanding job with heavy muscular activity have also been shown to strongly influence the disease progression [57]. The* number of syndesmophytes at the first presentation *is a well-known factor predicting progressive disease with damage [58]. The* CRP level at baseline *strongly correlates with disease progression [59]. Thus, it would appear that those who develop the disease recognised as axSpA* may *or* may not *progress from the stage where there is no visible structural damage* (nonradiographic)* towards the stage where there is structural damage visible as sacroiliitis (*radiographic *according to mNY criteria) on the standard radiograph. In older terminology, only the patients in latter category would be identified as* ankylosing spondylitis *but not those in the early stage. Again, the patients from this stage of the disease* may *or* may not *progress further towards the stage of new bone formation* (osteoproliferation)* visible as syndesmophytes and finally fusion of the vertebral bodies leading to the late radiographic stage called* “bamboo spine.”* These findings resulted in the concept of* “the continuum of axial SpA” *elegantly discussed by Rudwaleit et al. [60]. It is depicted in Figure 2. This figure focuses only on the axial component of SpA (axSpA) and shows it as a progressive pathology in the axial component of the body over time but only in some patients. The disease presents with a* chronic inflammatory back pain *of >3 months duration in persons <45 years of age. The course of the disease is unpredictable. In some patients, the disease may not progress beyond a certain stage on* the timeline*. In others, it may progress rapidly with the early development of radiographic changes with osteoproliferative and osteodestructive lesions. Such patients would be diagnosed as* ankylosing spondylitis *with mNY criteria. Yet in others, it may have a staccato start-stop course. Based upon the stage at which the patient is first seen, at an early stage (extreme left in time point) the sacroiliac joint may not show any abnormalities on a standard radiograph. Such patients are classified as* “nonradiographic axial SpA.”* Some of them may show active sacroiliitis on MRI (active osteitis by way of active bone marrow oedema). In some patients, the MRI may not be available (due to various reasons), and yet, clinically they have inflammatory back pain and other SpA features as described [60]. This subset of patients is classified as “nonradiographic axial SpA (nr-axSpA), clinical arm”; by definition all such patients would be HLA B27 +ve. On the other hand, patients where MRI is available, showing active sacroiliitis, are classified as “nonradiographic axial SpA (nr-axSpA), imaging arm.” Most of these nr-axSpA patients are also positive for HLA B27 but it is not essential for this subset of patients. Both of these above categories used to be identified as* “undifferentiated SpA”* in the past. Some patients may not progress further in the disease course on the timeline and remain at this stage throughout life. Others may continue to develop further anatomical damage in the sacroiliac joints over time (moving from left to right in time course) which would be detectable on the standard radiograph as sacroiliitis (according to mNY criteria, e.g., subcortical sclerosis, joint space narrowing, and erosions). These patients will now be identified as* radiographic axSpA *(r-axSpA). Some of these patients may not progress any further in time course and remain at this stage. Only some of these patients may further progress over time and develop* exuberant osteoproliferation *and* osteodestructive *lesions leading to the fusion of the sacroiliac joints and development of typical* syndesmophytes *visible on standard radiograph leading to* vertebral fusion *identified as* “bamboo spine.”*

**Figure 3:**
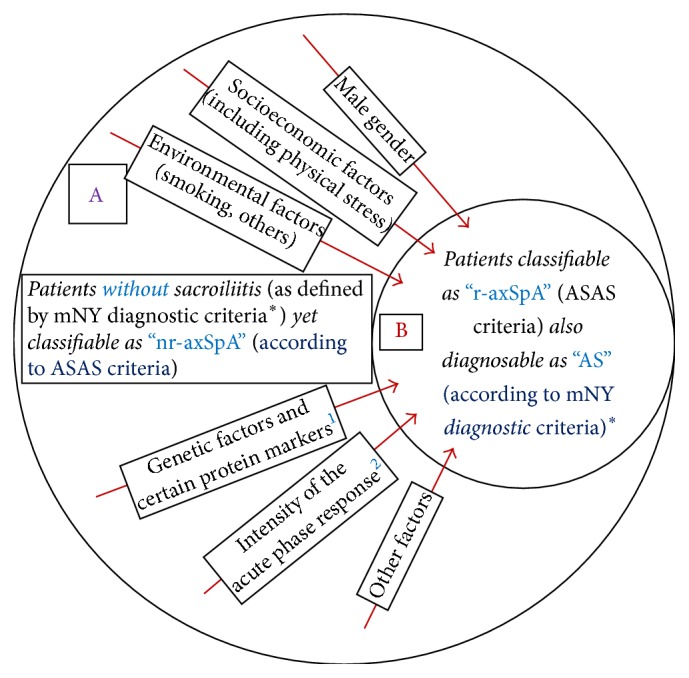
“nr-axSpA” = nonradiographic axial spondyloarthritis; “r-axSpA” = radiographic axial spondyloarthritis; ASAS = Assessment of SpondyloArthritis International Society; mNY diagnostic criteria = modified New York diagnostic criteria; “AS” = ankylosing spondylitis. mNY = modified New York, ASAS = Assessment of SpondyloArthritis International Society classification criteria; r-axSpA = radiographic axial spondyloarthritis; AS = ankylosing spondylitis. ^**∗**^It is to be noted that mNY are diagnostic criteria [10]. ^1^Genetic factors include HLA B27, ERAP-1, and IL23R and certain protein biomarkers include KIR3DL1, Sclerostin, and Dkk-1. ^2^Intensity of acute phase response is genetically determined, for example, C-reactive protein response.

Figure 3 shows the risk factors/forces* (as red arrows)* that lead to a progressive disease. Patients in the space “A” have inflammatory back pain but without structural damage in the SIJ that can be visualised on a standard radiograph. Patients in this stage are identified as having* nonradiographic axial SpA *(nr-axSpA). But, inflammation in these joints can be demonstrated with the use of MRI that would show subcortical bone marrow oedema confirming active sacroiliitis.* In many of these patients, the disease does not progress any further*. However, a proportion of patients, who may be exposed to certain risk factors (environmental, e.g., smoking, physically demanding job) or carry certain genetic factors (HLA B27, ERAP-1, IL23R) or are exposed to certain stochastic events, may get “pushed” from space “A” to enter space “B” and would show structural damage in the SIJ over time that would be visible on conventional radiograph as subarticular sclerosis, joint space narrowing, erosions, or joint fusion. These patients in space “B” would now be classified as* radiographic axSpA *(r-axSpA). In older terminology, such patients would be diagnosed as having ankylosing spondylitis (AS according to mNY criteria) [10]. Again, in some patients, the disease would not progress any further. But, in others, the risk factors/forces may continue to cause further structural changes. They develop* increasing osteoproliferation *visible as classical syndesmophytes causing fusion of the vertebral bodies identified as* “AS with bamboo spine.”* The risk factors/forces include male gender, socioeconomic factors (including physical stress), environment factors (smoking, others?), genetic factors (HLA B27, certain genotypes, ERAP-1, and IL23R), certain receptor protein biomarkers (KIR3DL1, Sclerostin, and Dkk-1, others?), intensity of acute phase response (genetically determined, CRP, others?), presence of syndesmophyte(s) at first presentation, and other possible factors.

## 6. The New Classification Criteria and Their Problems

In the search for an early and reliable diagnosis or classification criteria for AS, “*The Assessment of SpondyloArthritis International Society *(ASAS)” group, an international group of experts in the field of spondyloarthritis, took the lead. This group sought to propound entirely new classification criteria that would be sensitive as well as specific for accurate identification of patients with early AS at a stage before the appearance of radiographic sacroiliitis and osteoproliferation. After extensive studies and review of published reports, this group published the so-called ASAS classification criteria for SpA with the endeavour of detecting patients with early disease [11]. This new classification scheme for the first time used the term* radiographic *(r-axSpA; for those with definite sacroiliitis on a standard radiograph of the pelvis) and* nonradiographic *axial spondyloarthritis (nr-axSpA) for those with clinical features but without radiographic sacroiliitis. According to this classification,* the time-honoured term “AS” could be considered synonymous with “r-axSpA” *(Figure 2). The ASAS group emphasised that except for the absence of radiographic damage the nr-axSpA patients have a* comparable degree of clinical disease severity and disease burden to those who have r-axSpA *[22, 24].

The ASAS classification criteria, however, raised 2 obvious issues. The* first issue *was whether progression from nonradiographic to the radiographic stage is only a* question of disease duration *where every patient would develop radiographic changes if followed over a sufficient period. The* second issue *was related to the specificity of ASAS classification criteria for the early (nonradiographic) stage of the disease. The first issue that is progression of sacroiliac joint damage and osteoproliferation has already been discussed in a section above (“Inherent Difficulties in the Early Diagnosis of AS”). It is clear from the discussion on the long-term follow-up studies that the majority of the patients do progress from the nonradiographic to the radiographic stage over time. However, as shown in a widely quoted seminal paper by Professor Kumar et al., about the disease progression, there were 2 categories of patients [21]. The first category was of* 71.4% of the “unclassifiable” patients in 1983 who progressed and developed radiographic sacroiliitis (mNY criteria) 11 years later and could be diagnosed as AS*.* The second category included 28.5% patients who did not progress into AS (mNY criteria) or nr-axSpA stage*. Other workers have also reported similar findings; that is, at least about one-quarter of the patients with nonradiographic stage* did not progress *to the stage of radiographic sacroiliitis. It is to the credit of the ASAS group that they had not only stated such difficulties in the classification but also stated an important point that the disease progression seems to occur* only in predisposed individuals *[60]. The second issue of specificity of the ASAS classification criteria was answered in an elegant study from Leeds by Aydin's group [61]. The study showed that accurate recognition of real early stage of AS was difficult because after a period, some of those classifiable as nr-axSpA (the so-called early AS patients) turned out to have other unrelated cause(s) for back pain [61]. Independently, other workers also strongly objected to the notion that axial SpA is early AS [51].

There was another* (third)* problem related to the nr-axSpA category of the new ASAS classification criteria [62]. At the time the application to the Federal Drug Administration (FDA) of the USA was submitted for the approval of TNFi treatment in nr-axSpA patients, there were only 2 controlled trials in this group of patients [63, 64]. In these 2 studies, the FDA committee noted discrepancies in the reading of the radiographs with some degree of uncertainty in the classification of patients into radiographic and nonradiographic categories [62]. Also, as discussed earlier, questions were raised about the certainty of the progression of the disease to the radiographic stage [62]. These and a few additional issues have been the reason that the FDA withheld the approval for the use of TNFi in this class of patients. In a detailed discussion of this issue, the experts felt that the so-called “clinical SpA features” described in ASAS classification require a relook because of their nonspecificity [62, 65]. It is hoped that the classification criteria for nr-axSpA would soon be revised to make it more specific.

### 6.1. Is Nonradiographic SpA the Same as Ankylosing Spondylitis?

The above discussion makes it amply clear that SpA consists of a spectrum of related, severely painful inflammatory conditions of the spine, with a variable nonlinear course with periods of intense disease activity followed by periods of quiescence with no progression of damage. In some individuals, the disease may get arrested at an early stage without further progression and damage. In others, it may slowly progress with visible damage on radiograph with little or minimal osteoproliferation. In yet others, it may be progressive or rapidly progressive causing damage and osteoproliferation with the formation of syndesmophytes and “bamboo spine.” At each of these stages the disease shows a “stop-start” staccato course with phases of slow or rapid progression, then no progression at all for a variable time course only to restart progressing. As depicted in Figure 2, at the extreme left are the patients with symptoms of spinal pain, mostly in the lower back and buttocks with clinical features of an inflammatory back pain [13, 16, 17]. They would not show sacroiliitis on a standard radiograph of the SISI joints. Their symptoms could be anywhere from “very mild” to “very severe.”* In the past, this was identified as “unclassifiable” or “undifferentiated” SpA. Now, using ASAS classification criteria, these patients would be recognised as “nonradiographic SpA” *(nr-xSpA). In a proportion of them,* the disease may get arrested at this stage *without any further progress. Moving further in the time course,* some patients would progress *to the stage when, with fluctuating symptoms, the radiographs of their sacroiliac joints would start showing the presence of definite sacroiliitis. Before the publication of the ASAS classification, using the mNY criteria, these patients were diagnosed as having AS. The ASAS classification would now place them in the category of radiographic axial SpA (r-axSpA).* In a proportion of these patients, the disease would get arrested at this stage. *They may keep having fluctuating symptoms with disease flares. However, they may not progress to the stage of fusion of the vertebrae and may not show the presence of syndesmophytes.* A variable proportion of such patients, however, may progress further and develop an increasing number of syndesmophytes*. Their spinal movements would decrease steadily. Over time this category of patients would start showing the typical posture with hyperextension of the atlantoaxial joint, head protruding forward from the rest of the trunk, exaggerated upper dorsal kyphosis, loss of lumbar lordosis with decreased movement of the neck, decreased chest expansion, and restriction of the mobility of the lumbar spine in all the planes. Radiograph of the pelvis would show advanced sacroiliitis. The dorsolumbar spinal radiograph would show typical syndesmophytes in both lateral and the anterior-posterior view. This 3rd, and the last stage of axSpA is radiographically called* “bamboo spine.”* An artificial comparison between patients at the extreme left of the SpA “continuum” (Figure 2) with those at the extreme right with advanced structural damage is bound to show marked differences so much so that they may appear as different disease entities. Yet, all of them are from the same cohort with similar clinical features. The only difference is that some of them get* “selected”* to develop progressive damage due to stochastic factors (i.e., the risk factors/forces as discussed above) while other do not. Yet, as pointed out, the severity of symptoms and the “disease burden” is similar between these 2 groups of patients and they deserve similar treatment. Be that as it may, in general, r-axSpA classifiable with ASAS criteria can also be diagnosed as AS. But, strictly speaking, there could be a clinical scenario where the patient would not be classified as r-axSpA using the ASAS criteria, and yet, he/she can be diagnosed as “AS” with mNY diagnostic criteria. Thus, a patient who develops inflammatory back pain* after *the age of 45 years, with the SIJ radiograph showing significant sacroiliitis (according to MNY criteria), will be diagnosed as having AS. Yet,* the patient would not be classified as r-axSpA*. This is because at the “entry point” (or the stem) of the ASAS classification criteria* “age of onset < 45 years of age” *is essential by definition. This is one of the arguments against the use of the term “r-axSpA” and “AS” interchangeably. There could also be a situation which could be just the reverse of the above clinical example. Thus, there could be a patient with typical inflammatory back pain that started <45 years of age, the radiograph of the spine shows extensive syndesmophytes (“bamboo spine”), and yet the radiograph of the sacroiliac joints does not show significant sacroiliitis. Such a patient would be classifiable as “r-axSpA” but would not be diagnosed as having “AS” according to mNY diagnostic criteria.

## 7. Conclusion

*In simple terms, nr-axSpA and AS are different stages on the spectrum of a clinical entity called axSpA*. There are forces/risk factors that push patients towards the right-end of the spectrum shown in Figures 2 and 3. Those moving to the right of the spectrum are* distinguished entirely based upon increasing imaging abnormalities in the sacroiliac joints *and later on in spine. But, their clinical and laboratory abnormalities remain indistinguishable irrespective of their stage in the continuum of axSpA, depicted in Figure 2. Therefore, clinically such patients should be* simply diagnosed as having axSpA and treated for this disease*. Classification criteria are meant for research and clinical trials and, therefore, need to be highly specific. From this standpoint, the mNY criteria are strict and therefore highly specific with little chance of similar but different conditions getting included in this group. Therefore, if used in drug trials they would ensure that patients with back pain due to non-SpA conditions do not get included and mitigate the results. On the other hand, the ASAS classification criteria could have problems of specificity, as discussed by Deodhar et al. [62] and Robinson et al. [51]. Due to decreased specificity, there could be an issue of inclusion of conditions that are not within the spectrum of SpA. Therefore, they should be further refined to ensure detecting early disease with specificity that would be important in research and drug trials. Some of these issues have been recently discussed by Deodhar and colleagues [66].

## References

[1] Taurog J. D., Chhabra A., Colbert R. A. (2016). Ankylosing spondylitis and axial spondyloarthritis. *New England Journal of Medicine*.

[2] Khan M. A. (2002). Update on spondyloarthropathies. *Annals of Internal Medicine*.

[3] Malaviya A. N., Sawhney S., Mehra N. K., Kanga U. (2014). Seronegative arthritis in South Asia: an up-to-date review. *Current Rheumatology Reports*.

[4] Moll J. M., Haslock I., Macrae I. F., Wright V. (1974). Associations between ankylosing spondylitis, psoriatic arthritis, reiter*ʼ*s disease, the intestinal arthropathies, and behcet*ʼ*s syndrome. *Medicine (Baltimore)*.

[5] Wright V. (1978). Seronegative polyarthritis. a unified concept. *Arthritis and Rheumatism*.

[6] Dougados M., van der Linden S., Juhlin R. (1991). The European spondylarthropathy study group preliminary criteria for the classification of spondylarthropathy. *Arthritis and Rheumatism*.

[7] Braun J., Van der Heijde D., Dougados M. (2002). Staging of patients with ankylosing spondylitis: a preliminary proposal. *Annals of the Rheumatic Diseases*.

[8] Van Tubergen A., Weber U. (2012). Diagnosis and classification in spondyloarthritis: identifying a chameleon. *Nature Reviews Rheumatology*.

[9] Rothschild B., Feldtkeller E., Inman R., Sieper J. (2016). Spondyloarthritis in antiquity and in history. *Oxford Textbook of Axial Spondyloarthritis*.

[10] van der Linden S., Valkenburg H. A., Cats A. (1984). Evaluation of diagnostic criteria for ankylosing spondylitis. a proposal for modification of the New York criteria. *Arthritis and Rheumatism*.

[11] Rudwaleit M., van Der Heijde D., Landewé R. (2009). The development of assessment of SpondyloArthritis international society classification criteria for axial spondyloarthritis (part II): validation and final selection. *Annals of the Rheumatic Diseases*.

[12] van Gaalen F., van der Heijde D., Dougados M., Inman R., Sieper J. (2016). Diagnosis and classification of axial spondyloarthritis. *Oxford Textbook of Axial Spondyloarthritis*.

[13] Calin A., Porta J., Fries J. F., Schurman D. J. (1977). Clinical history as a screening test for ankylosing spondylitis. *The Journal of the American Medical Association*.

[14] Prakash S., Mehra N. K., Bhargava S., Malaviya A. N. (1983). HLA B27 related "unclassifiable" seronegative spondyloarthropathies. *Annals of the Rheumatic Diseases*.

[15] Amor B., Dougados M., Mijiyawa M. (1990). Criteres de classification des spondyloarthropathies. *Revue du Rhumatisme et des Maladies Ostéo-Articulaires*.

[16] Rudwaleit M., Metter A., Listing J., Sieper J., Braun J. (2006). Inflammatory back pain in ankylosing spondylitis: a reassessment of the clinical history for application as classification and diagnostic criteria. *Arthritis and Rheumatism*.

[17] Sieper J., van der Heijde D., Landewé R. (2009). New criteria for inflammatory back pain in patients with chronic back pain: a real patient exercise by experts from the Assessment of SpondyloArthritis international Society (ASAS). *Annals of the Rheumatic Diseases*.

[18] Gladman D. D. (2015). Editorial: what is peripheral spondyloarthritis?. *Arthritis and Rheumatology*.

[19] Stolwijk C., Essers I., van Tubergen A. (2015). The epidemiology of extra-articular manifestations in ankylosing spondylitis: a population-based matched cohort study. *Annals of the Rheumatic Diseases*.

[20] Mau W., Zeidler H., Mau R. (1988). Clinical features and prognosis of patients with possible ankylosing spondylitis. Results of a 10-year follow-up. *Journal of Rheumatology*.

[21] Kumar A., Bansal M., Srivastava D. N. (2001). Long-term outcome of undifferentiated spondylarthropathy. *Rheumatology International*.

[22] Boonen A., Sieper J., van der Heijde D. (2015). The burden of non-radiographic axial spondyloarthritis. *Seminars in Arthritis and Rheumatism*.

[23] Braun J., Bollow M., Neure L. (1995). Use of immunohistologic and in situ hybridization techniques in the examination of sacroiliac joint biopsy specimens from patients with ankylosing spondylitis. *Arthritis and Rheumatism*.

[24] Rudwaleit M., Listing J., Marker-Hermann E., Zeidler H., Braun J., Sieper J. (2004). The burden of disease in patients with ankylosing spondylitis (AS) and pre-radiographic axial spondyloarthritis. *Arthritis and Rheumatism*.

[25] http://www.webmd.com/rheumatoid-arthritis/news/19991110/fda-approves-remicade-for-treatment-of-rheumatoid-arthritis

[26] Marzo-Ortega H., McGonagle D., O'Connor P., Emery P. (2001). Efficacy of etanercept in the treatment of the entheseal pathology in resistant spondylarthropathy: a clinical and magnetic resonance imaging study. *Arthritis and Rheumatism*.

[27] Braun J., Brandt J., Listing J. (2002). Treatment of active ankylosing spondylitis with infliximab: a randomised controlled multicentre trial. *The Lancet*.

[28] van Den Bosch F., Kruithof E., Baeten D. (2002). Randomized double-blind comparison of chimeric monoclonal antibody to tumor necrosis factor *α* (infliximab) versus placebo in active spondylarthropathy. *Arthritis and Rheumatism*.

[29] Gorman J. D., Sack K. E., Davis J. C. (2002). Treatment of ankylosing spondylitis by inhibition of tumor necrosis factor *α*. *New England Journal of Medicine*.

[30] Maksymowych W. P., Jhangri G. S., Lambert R. G. (2002). Infliximab in ankylosing spondylitis: a prospective observational inception cohort analysis of efficacy and safety. *Journal of Rheumatology*.

[31] Breban M., Vignon E., Claudepierre P. (2002). Efficacy of infliximab in refractory ankylosing spondylitis: results of a six-month open-label study. *Rheumatology*.

[32] Brandt J., Khariouzov A., Listing J. (2003). Six-month results of a double-blind, placebo-controlled trial of etanercept treatment in patients with active ankylosing spondylitis. *Arthritis and Rheumatism*.

[33] Davis J. C., van Der Heijde D., Braun J. (2003). Recombinant human tumor necrosis factor receptor (etanercept) for treating ankylosing spondylitis: a randomized, controlled trial. *Arthritis and Rheumatism*.

[34] Braun J., Baraliakos X., Golder W. (2003). Magnetic resonance imaging examinations of the spine in patients with ankylosing spondylitis, before and after successful therapy with infliximab: evaluation of a new scoring system. *Arthritis and Rheumatism*.

[35] Oostveen J. C. M., Prevo R. L., den Boer J. A., Van De Laar M. A. F. J. (1999). Early detection of sacroiliitis on magnetic resonance imaging and subsequent development of sacroiliitis on plain radiography. A prospective, longitudinal study. *Journal of Rheumatology*.

[36] Ahlström H., Feltelius N., Nyman R., Hällgren R. (1990). Magnetic resonance imaging of sacroiliac joint inflammation. *Arthritis and Rheumatism*.

[37] Braun J., Bollow M., Eggens U., König H., Distler A., Sieper J. (1994). Use of dynamic magnetic resonance imaging with fast imaging in the detection of early and advanced sacroiliitis in spondylarthropathy patients. *Arthritis and Rheumatism*.

[38] Rudwaleit M., Jurik A. G., Hermann K. G. A. (2009). Defining active sacroiliitis on magnetic resonance imaging (MRI) for classification of axial spondyloarthritis: a consensual approach by the ASAS/OMERACT MRI group. *Annals of the Rheumatic Diseases*.

[39] Sieper J., Rudwaleit M. (2005). How early should ankylosing spondylitis be treated with tumour necrosis factor blockers?. *Annals of the Rheumatic Diseases*.

[40] Arends S., van Der Veer E., Kallenberg C. G. M., Brouwer E., Spoorenberg A. (2012). Baseline predictors of response to TNF-*α* blocking therapy in ankylosing spondylitis. *Current Opinion in Rheumatology*.

[41] van Der Linden S. M., Valkenburg H. A., De Jongh B. M., Cats A. (1984). The risk of developing ankylosing spondylitis in HLA-B27 positive individuals. a comparison of relatives of spondylitis patients with the general population. *Arthritis and Rheumatism*.

[42] Said-Nahal R., Miceli-Richard C., Berthelot J.-M. (2000). The familial form of spondylarthropathy: a clinical study of 115 multiplex families. *Arthritis and Rheumatism*.

[43] Hart F. D., Robinson K. C. (1959). Ankylosing spondylitis in women. *Annals of the Rheumatic Diseases*.

[44] Gracey E., Yao Y., Green B. (2016). Sexual dimorphism in the th17 signature of ankylosing spondylitis. *Arthritis and Rheumatology*.

[45] Kiltz U., Baraliakos X., Karakostas P. (2012). Do patients with non-radiographic axial spondylarthritis differ from patients with ankylosing spondylitis?. *Arthritis Care and Research*.

[46] Wallis D., Haroon N., Ayearst R., Carty A., Inman R. D. (2013). Ankylosing spondylitis and nonradiographic axial spondyloarthritis: part of a common spectrum or distinct diseases?. *Journal of Rheumatology*.

[47] Malaviya A. N., Kalyani A., Rawat R. (2015). Is radiographic axial SpA a distinct subset with more severe axial involvement? Comment on the article by Deodhar et al.. *Arthritis and Rheumatology*.

[48] Malaviya A. N., Kalyani A., Rawat R., Gogia S. B. (2015). Comparison of patients with ankylosing spondylitis (AS) and non-radiographic axial spondyloarthritis (nr-axSpA) from a single rheumatology clinic in New Delhi. *International Journal of Rheumatic Diseases*.

[49] Jeong H., Yoon J. Y., Park E.-J. (2015). Clinical characteristics of nonradiographic axial spondyloarthritis in Korea: a comparison with ankylosing spondylitis. *International Journal of Rheumatic Diseases*.

[50] Baraliakos X., Braun J. (2015). Non-radiographic axial spondyloarthritis and ankylosing spondylitis: what are the similarities and differences. *Rheumatic Musculoskeletal Diseases Open*.

[51] Robinson P. C., Wordsworth B. P., Reveille J. D., Brown M. A. (2013). Axial spondyloarthritis: a new disease entity, not necessarily early ankylosing spondylitis. *Annals of the Rheumatic Diseases*.

[52] Tsui F. W. L., Tsui H. W., Akram A., Haroon N., Inman R. D. (2014). The genetic basis of ankylosing spondylitis: new insights into disease pathogenesis. *Application of Clinical Genetics*.

[53] Vendelbosch S., Heslinga S. C., John M. (2015). Study on the protective effect of the KIR3DL1 gene in ankylosing spondylitis. *Arthritis and Rheumatology*.

[54] Ustun N., Tok F., Kalyoncu U. (2014). Sclerostin and Dkk-1 in patients with ankylosing spondylitis. *Acta Reumatologica Portuguesa*.

[55] Pradeep D. J., Keat A., Gaffney K. (2008). Predicting outcome in ankylosing spondylitis. *Rheumatology*.

[56] Ward M. M., Hendrey M. R., Malley J. D. (2009). Clinical and immunogenetic prognostic factors for radiographic severity in ankylosing spondylitis. *Arthritis Care and Research*.

[57] Ramiro S., Landewé R., van Tubergen A. (2015). Lifestyle factors may modify the effect of disease activity on radiographic progression in patients with ankylosing spondylitis: a longitudinal analysis. *Rheumatic Musculoskeletal Diseases Open*.

[58] Mandl P., Navarro-Compán V., Terslev et al. L. (2015). EULAR recommendations for the use of imaging in the diagnosis and management of spondyloarthritis in clinical practice. *Annals of the Rheumatic Diseases*.

[59] Braun J., Baraliakos X., Hermann K. A., Xu S., Hsu B. (2016). Serum C-reactive protein levels demonstrate predictive value for radiographic and magnetic resonance imaging outcomes in patients with active ankylosing spondylitis treated with Golimumab. *The Journal of Rheumatology*.

[60] Rudwaleit M., Khan M. A., Sieper J. (2005). Commentary: the challenge of diagnosis and classification in early ankylosing spondylitis: do we need new criteria?. *Arthritis and Rheumatism*.

[61] Aydin S. Z., Maksymowych W. P., Bennett A. N., McGonagle D., Emery P., Marzo-Ortega H. (2012). Validation of the ASAS criteria and definition of a positive MRI of the sacroiliac joint in an inception cohort of axial spondyloarthritis followed up for 8 years. *Annals of the Rheumatic Diseases*.

[62] Deodhar A., Reveille J. D., Van Den Bosch F. (2014). The concept of axial spondyloarthritis: joint statement of the spondyloarthritis research and treatment network and the assessment of spondyloarthritis international society in response to the US food and drug administration's comments and concerns. *Arthritis and Rheumatology*.

[63] Sieper J., van der Heijde D., Dougados M. (2013). Efficacy and safety of adalimumab in patients with non-radiographic axial spondyloarthritis: results of a randomised placebo-controlled trial (ABILITY-1). *Annals of the Rheumatic Diseases*.

[64] Landewé R., Braun J., Deodhar A. (2014). Efficacy of certolizumab pegol on signs and symptoms of axial spondyloarthritis including ankylosing spondylitis: 24-week results of a double-blind randomised placebo-controlled Phase 3 study. *Annals of the Rheumatic Diseases*.

[65] Yim S. Axial spondyloarthritis: regulatory considerations on its use as an indication for drug development. http://www.fda.gov/downloads/advisorycommittees/committeesmeetingmaterials/drugs/arthritisadvisorycommittee/ucm366488.pdf.

[66] Deodhar A., Strand V., Kay J., Braun J. (2016). The term “non-radiographic axial spondyloarthritis” is much more important to classify than to diagnose patients with axial spondyloarthritis. *Annals of the Rheumatic Diseases*.

